# Prevalence and Antimicrobial Resistance of Microbes Causing Bloodstream Infections in Unguja, Zanzibar

**DOI:** 10.1371/journal.pone.0145632

**Published:** 2015-12-23

**Authors:** Annette Onken, Abdulrahman K. Said, Melissa Jørstad, Pål A. Jenum, Bjørn Blomberg

**Affiliations:** 1 Department of Medical Microbiology, Vestre Viken Health Trust, Drammen, Norway; 2 Department of Medicine, Mnazi Mmoja Hospital, Zanzibar, Tanzania; 3 Department of Medicine, Haukeland University Hospital, Bergen, Norway; 4 National Center for Tropical Infectious Diseases, Haukeland University Hospital, Bergen, Norway; 5 Department of Clinical Sciences, University of Bergen, Bergen, Norway; Second University of Naples, ITALY

## Abstract

**Background:**

Bloodstream infections (BSI) are frequent and cause high case-fatality rates. Urgent antibiotic treatment can save patients’ lives, but antibiotic resistance can render antibiotic therapy futile. This study is the first to collect epidemiological data on BSI from Unguja, Zanzibar.

**Methods:**

Clinical data and blood for culturing and susceptibility testing of isolated microbes were obtained from 469 consecutively enrolled neonates, children and adults presenting with signs of systemic infections at Mnazi Mmoja Hospital (MMH), Zanzibar.

**Results:**

Pathogenic bacteria were recovered from the blood of 14% of the patients (66/469). The most frequently isolated microbes were *Klebsiella pneumoniae*, *Escherichia coli*, *Acinetobacter* spp. and *Staphylococcus aureus*. Infections were community-acquired in 56 patients (85%) and hospital-acquired in 8 (12%) (data missing for 2 patients). BSI caused by extended-spectrum beta-lactamase (ESBL) producing *Enterobacteriaceae* (*E*. *coli*, *K*. *pneumoniae*) was found in 5 cases, of which 3 were community-acquired and 2 hospital-acquired. Three of these patients died. Six of 7 *Salmonella* Typhi isolates were multidrug resistant. *Streptococcus pneumoniae* was found in one patient only.

**Conclusions:**

This is the first report of ESBL-producing bacteria causing BSI from the Zanzibar archipelago. Our finding of community-acquired BSI caused by ESBL-producing bacteria is alarming, as it implies that these difficult-to-treat bacteria have already spread in the society. In the local setting these infections are virtually impossible to cure. The findings call for increased awareness of rational antibiotic use, infection control and surveillance to counteract the problem of emerging antimicrobial resistance.

## Introduction

Sepsis is a major health problem associated with high mortality rates [1,2]. Data on both mortality and incidence of sepsis in Africa are limited. A mean mortality rate of 18.1% is reported in a meta-analysis on community acquired bloodstream-infection (BSI) in Africa [1]. In a study on a pediatric population in Tanzania a mortality rate of 34.9% was found [3]. High prevalence of immunosuppression due to malnutrition and other infectious diseases including human immunodeficiency virus (HIV) infection and measles may contribute to an increased burden of severe bacterial infections in African countries [3,4]. BSI caused by multidrug-resistant, extended-spectrum beta-lactamase (ESBL) producing Gram-negative bacilli is associated with very high case-fatality rates approaching those of the pre-antibiotic era [3]. Epidemiological data from specific geographic regions are needed to optimize guidelines for empirical treatment. In Zanzibar, data on the etiology of BSI have only been published from Pemba, the less populated of the two main islands comprising Zanzibar [5]. We performed a prospective cohort study in patients suspected of having BSI at Mnazi Mmoja Hospital (MMH) on Unguja, the most populated island of the Zanzibar archipelago, Zanzibar, Tanzania. The aim was to identify the most common bacterial pathogens causing BSI and to determine their antimicrobial susceptibility.

## Material and Methods

### Study site

Mnazi Mmoja hospital (MMH), Zanzibar, Tanzania, is the main referral and teaching hospital of the Zanzibar Archipelago with a population estimated to about 1.3 million in 2012 (http://www.nbs.go.tz). The hospital also offers primary and secondary health care for the residents of Zanzibar city with a population of about 600,000. The hospital has 544 beds.

### Study design

Patients in the medical, pediatric and neonatal departments were enrolled in the study if they, either on admission or during their hospital stay, had fever (≥ 38.3°C in adults, ≥ 38.5°C in children) or hypothermia (<36.0°C), tachypnoea >20/min, tachycardia >90/min or were otherwise suspected to have systemic bacterial infection as judged by the clinician. Demographic and clinical information was obtained. Infections were defined as community-acquired and hospital-acquired, if pathogens were detected in samples taken within 2 days after admission and > 2 days after admission, respectively.

### Methods

Patients were included over a period of 7 months (26^th^ March to 22^nd^ June 2012, 26^th^ October to 21^st^ December 2012, and 4^th^ February to 22^nd^ April 2013). Blood specimens were inoculated in BACTEC Myco/F lytic blood culturing vials (Becton Dickinson, Franklin Lakes, N.J.), one bottle per episode of febrile illness. The bottles were incubated at 37°C for 7 days and checked daily on Monday to Friday and once on either Saturdays or Sundays for microbial growth by inspecting the bottom indicator of the bottle for fluorescence [6].

Positive samples were examined by microscopy of Gram-stained preparations and sub-cultivated for two days on chocolate agar and on human blood agar in 5% CO_2_, and on MacConkey agar in aerobe atmosphere. The isolates were identified according to established conventional procedures [7]. Samples with polymicrobial growth were included, if at least one of the microbes was considered a pathogen. As most patients had only one blood culture drawn, it was not possible to ascertain the role of bacteria of uncertain clinical relevance, such as coagulase-negative staphylococci, diphtheroids and *Bacillus* species. Thus, these species were considered contaminants.

Gram-negative rods were identified using standard biochemical tests and the API 20E or the API 20NE system (bioMérieux, Marcy-l’Etoile, France). Susceptibility testing was performed by disc diffusion technique as described in the EUCAST guidelines [8]. Reports on the results were sent to the wards.

Isolates of pathogenic microbes were sent to Bærum Hospital, Vestre Viken Health Trust, Norway, for quality control and further identification by VITEK 2 and, if necessary, matrix-assisted laser desorption/ionization time-of-flight (MALDI-TOF) mass spectrometry and/or 16S rDNA polymerase chain reaction (PCR) sequencing (performed at Oslo University Hospital and/or the Norwegian Institute of Public Health, Oslo, Norway). In Norway, susceptibility testing was performed by disc diffusion technique and/or Etest gradient system (bioMérieux) according to the EUCAST guidelines and/or by VITEK 2 and interpreted by the S-I-R system [9]. *Enterobacteriaceae* isolates resistant to cefotaxime or ceftazidime, were further assessed for ESBL type resistance by ESBL Etest gradient system (bioMérieux, France), ESBL CTX-M in-house PCR [10] and AmpC in-house PCR [11].

### Statistical analysis and ethical approval

Differences between proportions were compared using Fisher’s exact test with cutoff for statistical significance at p = 0.05. The research protocol was approved by the Zanzibar Medical Research and Ethical Committee (ZAMREC), record no ZAMREC /0004/JAN/012, and by the Regional Committee for Medical Research Ethics Health Region West (REK III), Norway, record no 201124397/2011/2439/REK vest. Written informed consent was obtained from the patient or, in the case of children, from a parent or a responsible family member.

## Results

### Microbes

A total of 470 blood culture samples from 469 patients (242 male, 226 female, 1 with gender not reported) were consecutively included in the study, including 148 (31%) from children (aged >1 month to 5 years of age), and 113 (24%) from neonates (age ≤ 1 month) (Table 1). One blood culture bottle per patient was taken except for one patient who had two blood cultures taken 3 weeks apart, because of a new episode of febrile illness. Pathogenic microbes were isolated from 14.0% (66/470) of the blood cultures (66 patients), including 15 cultures with growth of multiple isolates, out of which at least one pathogen. The most frequent isolated pathogens were *K*. *pneumoniae* (n = 11), *E*. *coli* (n = 10), *Acinetobacter* spp. (n = 10) and *S*. *aureus* (n = 9) (Tables 2 and 3). A further 53 (11%) blood cultures yielded growth of bacteria considered to be probable contaminants: coagulase-negative staphylococci (n = 46, 10%), diphtheroids (n = 2), *Bacillus* sp. (n = 1), Gram-positive rods (n = 3), and mixture of coagulase negative staphylococcus and diphtheroids (n = 1).

**Table 1:** Distribution of 469 patients by gender and age group.

	Neonate* (≤ 1 month)	<5 years (>1 month to <5 years)	5–15 years	Adults
**Male**	57	84	25	76
**Female**	55	64	15	92
**Total**	113	148	40	168

*1 patient no info on gender.

**Table 2:** Frequency of bacterial and fungal pathogens causing community- and hospital-acquired bloodstream-infections in patients admitted at Mnazi Mmoja Hospital, Zanzibar.

Pathogens	Total	Commu-nity acquired**	Hospital acquired***	Missing data
**Total**	**79 (100%)**	**67 (100%)**	**10 (100%)**	**2**
**Total Gram-negative bacteria** ****	**59 (75%)**	**51 (76%)**	**7 (70%)**	**1**
Total *Enterobacteriaceae*	**36**	**31**	**4**	**1**
*-E*.*coli* *	**10**	**9**	**1**	
*-K*. *pneumoniae* *	**11**	**8**	**3**	
*-Salmonella serovar* Typhi*	**7**	**6**		**1**
*-Enterobacter cloacae* *	**4**	**4**		
*-Enterobacter aerogenes*	**1**	**1**		
*-Proteus mirabilis*	**2**	**2**		
*-Citrobacter freundii*	**1**	**1**		
Total non-*Enterobacteriaceae*	**19**	**17**	**2**	
*-Pseudomonas putida*	**1**	**1**		
*-Acinetobacter baumannii* *	**6**	**6**		
*-Acinetobacter* spp. non- *baumannii*	**4**	**3**	**1**	
*-Ochrobactrum anthropi*	**3**	**2**	**1**	
*-Achromobacter* spp.	**5**	**5**		
Unidentified Gram-negative rods	**4**	**3**	**1**	
**Total Gram-positive bacteria**	**18 (23%)**	**14 (21%)**	**3 (30%)**	**1**
*Staphylococcus aureus*	**9**	**7**	**1**	**1**
*Enterococcus faecalis*	**4**	**3**	**1**	
*Enterococcus faecium*	**1**		**1**	
*Streptococcus pneumoniae* *	**1**	**1**		
Group B *streptococcus*	**2**	**2**		
*Rhodococcus equi*	**1**	**1**		
**Total yeast**	**2 (2%)**	**2 (3%)**		
*Candida guillermondii*	**1**	**1**		
Other yeast	**1**	**1**		

*Isolates not stored in Zanzibar: *E*. *coli* 3, *K*. *pneumoniae* 2, *S*. Typhi 1, *A*. *baumannii* 2, unidentified gram-negative rods 2, *S*. *aureus* 2.

Isolates that succumbed during transport to Norway: *E*. *coli* 2, *K*. *pneumoniae* 1, unidentified gram-negative rod 1, *S*. *pneumoniae* 1.

6 strains of *S*. Typhi and 1 unidentified gram-negative rod (possible Brucella) sent to Norway were inactivated because of transport regulations.

** Community-acquired infection, i.e. blood-culture obtained ≤ 48 hours from of admission.

***Hospital-acquired infection: Blood-culture obtained > 48 hours from admission.

****The percentage refers to the proportion of all pathogenic bacterial and fungal isolates.

**Table 3:** Frequency of bacterial and fungal pathogens causing bloodstream infection in among patients admitted to Mnazi Mmoja Hospital, Zanzibar, by age groups.

Pathogen	Total	Neonate (≤1 month)	<5 years (>1 month to <5 years)	5–15 years	Adult
**Total**	**79**	**43 (54%)**	**10 (13%)**	**8 (10%)**	**18 (23%)**
**Total Gram-negatives**	**59**	**33**	**9**	**4**	**13**
Total *Enterobacteriaceae*	**36**	**19**	**5**	**3**	**9**
*-E*.*coli*	**10**	**6**	**1**	**1**	**2**
*-K*. *pneumoniae*	**11**	**8**	**1**		**2**
*-Salmonella serovar* Typhi	**7**		**1**	**2**	**4**
-Other *Enterobacteriaceae*	**8**	**5**	**2**		**1**
Total non-*Enterobacteriaceae*	**19**	**11**	**4**	**1**	**3**
Unidentified Gram-negative rods	**4**	**3**			**1**
**Total Gram-positives**	**18**	**10**		**4**	**4**
*S*. *aureus*	**9**	**3**		**4**	**2**
*Enterococcus faecalis*	**4**	**4**			
*Enterococcus faecium*	**1**	**1**			
*S*. *pneumoniae*	**1**				**1**
Group B *streptococcus*	**2**	**1**			**1**
*Rhodococcus equi*	**1**	**1**			
**Total yeast**	**2**		**1**		**1**

Among the 66 patients with pathogenic microbes in the blood culture, 56 patients (85%) had community-acquired infection, and 8 patients (12%) had hospital-acquired infection. Mode of acquisition could not be assessed for 2 patients due to missing information.

Eighteen isolates of pathogens could not be retested in Norway as they either were not stored in the freezer in Zanzibar (n = 12) or did not survive the transport (n = 6).

### Antimicrobial resistance (Table 4)

**Table 4:** Antimicrobial susceptibility (number tested) of *Enterobacteriaceae* isolates causing bloodstream infection in patients admitted to Mnazi Mmoja Hospital, Zanzibar.

	*K*. *pneumoniae* (n = 11)	*E*. *coli* (n = 10)	*S*. Typhi (n = 7)	other *Entero-bacteriaceae* (n = 8)	Total *Entero-bacteriaceae* (n = 36)*	Total susceptibility percentage
**Ampicillin**	0**	6	0	1	7	19%
**Cefotaxime**	6	9	7	7	29	81%
**Piperacillin/tazobactam**	7	10		8	25	86%
**Meropenem**	10***	10	7	8	35	97%
**Gentamicin**	8	10		8	26	90%
**Ciprofloxacin**	10	9	6	8	33	92%
**Trimethoprim/sulfamethoxazole**	6	3	0	5	14	39%

*for Piperacillin/tazobactam only 29 isolates were analysed. For *S*. Typhi, the susceptibility to gentamicin was not analyzed: aminoglycosids are not recommended for treatment of *S*. Typhi infections because they lack activity against intracellular Salmonella[12].

**K. *pneumoniae* are naturally resistant to ampicillin.

***one intermediate to meropenem, only tested in Zanzibar, cefotaxime, ampicillin, trimethoprim-sulfamethoxazole R, probable ESBL positive.

Six isolates (from 5 patients) of *Enterobacteriaceae* (*Klebsiella pneumoniae* (5) and *E*. *coli* (1)) were suspect of ESBL-production as they displayed resistance to cefotaxime or/and ceftazidime on disc-diffusion testing. ESBL-Etest was positive for 4 isolates (from 3 patients), *K*. *pneumoniae* (3) and *E*. *coli* (1). For 2 of the *K*. *pneumoniae* isolates including 1 isolate testing intermediate for meropenem, the results on further testing for ESBL production are lacking.

The 4 ESBL E-test positive isolates were tested with PCR for CTX-M genotype. Three of the isolates were CTX-M PCR positive. The fourth isolate (*K*. *pneumoniae*) was CTX-M PCR negative, but CMY-PCR positive (belonging to the AmpC β-lactamases).

Among the 5 patients with BSI caused by bacteria with confirmed or probable ESBL-production, the infection was classified as hospital-acquired in 2 patients and community-acquired in 3 patients. In 13 patients, ESBL-negative *E*. *coli* or *K*. *pneumoniae* or both (two patients had mixed infection with both *E*.*coli* and *K*. *pneumoniae*) were isolated, of which the majority were community-acquired (10/13). Three of the 5 (60%) patients with infection caused by confirmed or probable ESBL-positive bacteria died. Four of the 11 (36%) of the patients with infection caused by bacteria without ESBL-production died (data were missing for 2 patients). This difference in case-fatality rates was not statistically significant (p = 0.6).

All confirmed and probable ESBL-positive isolates were resistant to trimethoprim-sulfamethoxazole, while rates of co-resistance to other antibiotics were 3/5 to gentamicin, 2/5 to ciprofloxacin, 1/5 to piperacillin-tazobactam. No isolate displayed phenotypic carbapenem resistance. The majority of the *S*. Typhi isolates 6/7 (86%) were multidrug-resistant (i.e. resistant to ampicillin, trimethoprim-sulfamethoxazole and chloramphenicol), but susceptible to cefotaxime.

Only one isolate of *S*. *pneumoniae* was recovered. Resistance to oxacillin indicated a reduced susceptibility to penicillin G. Further testing was not possible as the isolate died. The only *Enterococcus faecium* that was isolated was high level gentamicin resistant, but susceptible to vancomycin. All nine *S*. *aureus* strains were susceptible to cefoxitin, clindamycin and erythromycin. No methicillin-resistant *S*. *aureus* (MRSA) was found. All 7 *S*. *aureus* isolates tested for pencillinase-production were positive, 2/9 were resistant to trimethoprim-sulfamethoxazole, 1/9 to tetracycline.

## Discussion

While ESBL producing microbes in clinical samples including blood cultures have been reported from other parts of the African continent [13], including the mainland of Tanzania [14–16], this is the first report of ESBL-producing bacteria causing bloodstream infections from the Zanzibar archipelago. In a recent study on bacteremia from the neighbor island Pemba [5] no ESBL-positive bacteria were found. The finding of ESBL-positive microbes in blood culture is associated with increased mortality [3]. We did not find significantly higher case-fatality rate in patients with bloodstream-infections caused by ESBL producing *Enterobacteriaceae* (3/5) compared to those caused by ESBL negative microbes (4/11), but the numbers of the patients were small.

Differences between the findings of the studies from MMH/ Unguja and Pemba in both etiology of bloodstream infections and the susceptibility patterns of the isolated microbes may be explained by the fact that Unguja has a more urban infrastructure and people have easier access to antimicrobials. Furthermore, Unguja has more extensive contact with mainland Tanzania, where a high prevalence of resistant microbes has been documented [15,16], and also more exposure to tourists and international travelers. These differences may imply a higher rate of preadmission antimicrobial treatment, although we have no evidence to support this.

While the study from Pemba assessed community-acquired infections at three district hospitals, our study was performed at an urban referral hospital and included nosocomial infections and patients in a neonatal intensive care unit. This may have led to selection of more severely ill patients, infections with more resistant microbes, and more frequent use of broad-spectrum antimicrobials, which in turn may also have contributed to the higher rate of resistant bacteria in our study.

Previously, ESBL-producing bacteria were largely associated with nosocomial infections, but according to more recent studies, infections caused by community-acquired ESBL-producing bacteria are increasing [17]. ESBL-positive bacteria at MMH were found not only in hospital-acquired, but also in community-acquired infections. Our finding of community-acquired bloodstream infections caused by ESBL producing bacteria is alarming, as it implies that these difficult-to-treat bacteria have already spread in the society. Treatment of infections caused by ESBL-producing bacteria is much more costly, if at all available, and leads to prolonged hospital stays for those who survive [18,19].

ESBL-positive bacteria are resistant to third generation cephalosporins. These are often used as first line medication in sepsis at MMH. ESBL resistance is plasmid-mediated. These plasmids also often carry resistance genes to other groups of antibiotics [20]. Therefore carbapenems are the cornerstone of treatment of infections caused by ESBL-producing bacteria. However, these antibiotics are expensive and generally not available in resource-constrained settings such as Zanzibar, rendering such infections virtually untreatable in the local setting. Even if carbapenems were available, their use in the absence of accessible microbiological diagnostic services is problematic. Low treatment success due to high prevalence of infections caused by resistant bacteria likely results in increasing empiric use of broad-spectrum antibiotics, which exerts a strong selection pressure favoring further emergence of multidrug-resistant bacteria in the hospital and the society. Infections caused by carbapenem-resistant bacteria have already been documented in nearby Dar es Salaam, in mainland Tanzania [21]. Globally, antimicrobial resistance to Gram-negative microbes is rising faster than in Gram-positive bacteria and there are no new antibiotics effective against Gram-negative bacteria in the immediate pipeline [22]. In countries with limited resources, the rapid emergence of antibiotic-resistant bacteria is furthermore promoted by patient overcrowding, overwhelmed health-care workers, limited hospital infrastructure, poor compliance with hand hygiene, and, lack of infection control programs [23]. Improved microbiological diagnosis, antibiotic susceptibility testing and epidemiological studies, may help guide sustainable, rational antibiotic use.

Comparison of the etiology of sepsis among different studies in Africa [1] is challenging, as different populations are included (adults, children, neonates, all age groups, community-acquired or/and nosocomial infections). The varying prevalence of other diseases, such as HIV-infections and malaria probably also have an impact on the findings [24], as well as the geographical region and the socio-economic structure. Our study population consists of all age groups, with both community-acquired and nosocomial infection, from an area with low prevalence of malaria and HIV-infection [25–27].

The prevalence of bacteremia in our study (14%) is in line with findings of a meta-analysis on the cause of community-acquired bloodstream infections in Africa, which found a prevalence of 13.4% among patients with fever [1]. *Salmonella enterica*, of which 41% were *Salmonella* Typhi, followed by *Streptococcus pneumoniae* were the most frequent isolated microbes, with *S*. *enterica* being the most common isolate in adults, and *S*. *pneumoniae* the most frequent in children. Other common bacteria were *S*. *aureus* and *E*. *coli*. [1]. We found only one isolate of *S*. *pneumoniae*, and we suspect, as in the study from Dar es Salaam [3], that frequent pre-hospital antibiotic use may have precluded the recovery of pneumococci from blood cultures, resulting in underestimation of the proportion of pneumococcal infections.

Multi-resistance in *S*. Typhi (resistant to ampicillin, cotrimaxozole and chloramphenicol) was more frequent in our study (86%, n = 6/7) than reported from Pemba (42%, n = 19/45, p = 0.046, Fisher’s exact test) [5]. Low and similar rates of resistance to ciprofloxacin were found at both sites.

The only *S*. *pneumoniae* in our study was oxacillin resistant implicating a decreased sensitivity to penicillin G. In the study from Pemba, *S*. *pneumoniae* was the second most common microbe (15%), after S. Typhi (58%), and 25% (3/12) of the pneumococcal isolates were penicillin resistant [5]. We found low rates of resistance among Gram-positive bacteria. No methicillin-resistant *S*. *aureus* (MRSA) was isolated. This is in line with the study from Pemba, but contrary to findings from other African countries and mainland Tanzania [3,28,29].

Non-fermentative gram-negative rods were frequently isolated from neonatal patients and must be regarded as real pathogens [30] as the immune system in neonates is still immature. *Acinetobacter* has been shown to cause severe disease, particularly in tropical countries [30]. The study from Dar es Salaam also found a high proportion of BSI attributable to non-fermenters (11.6%, 34/294) [3]. Non-fermentative gram-negative bacteria are generally isolated more frequently in sepsis especially in patients with underlying diseases [31]. However, contamination must also be considered in these cases.

Polymicrobial infection, i.e. growth of 2 or 3 different microbes from the same blood culture, occurred frequently (19% of BSIs) in our study, as in the study from Dar es Salaam (12%) [3]. Polymicrobial BSI may have been caused by translocation from gastrointestinal focus of infection, possibly in very sick or immunocompromised patients. Contamination of the samples may be another possible explanation. Better staff training in the technique of taking samples can reduce the risk of contamination.

The main limitation of the study is the low number of patients included. Another limitation is that only one sample per patient was taken, due to limited resources. Coagulase-negative staphylococci were therefore counted as contaminants, although they may have had clinical significance in some cases of immune-compromised patients or patients with indwelling devices and among neonates. No anaerobe culture was performed. Data were lacking on pre-treatment with antibiotics and outcome. As the study did not cover all seasons, possible seasonal variations may have been missed. Limitations in laboratory facilities and transport caused loss of some data [32,33].

## Conclusions

This is the first study of bloodstream-infections from Unguja, Zanzibar, and the first to document the presence of ESBL-producing multidrug-resistant *Enterobacteriaceae* as a cause of bloodstream infections in the Zanzibar archipelago. These infections are difficult to treat in the local setting and were associated with a high case-fatality rate. The finding of community-acquired infections caused by ESBL-resistant bacteria in Zanzibar is particularly worrying, as it indicates a general spread of these resistant bacteria in the society. The study findings call for prudent antibiotic use and focus on infection control in health-care settings.

More data are needed on the etiology and antimicrobial susceptibility in bloodstream infections in Zanzibar including the prevalence of multidrug-resistant ESBL-producing bacteria, and this knowledge can be used to guide the development of new treatment guidelines for MMH and Zanzibar. The education of health workers in rational use of antimicrobials as well as in infection control should be intensified.

## Supporting Information

S1 Table Data on study participants. (XLS)

S2 Table Data on bacterial isolates. (XLS)

## Abbreviations

BSI: Bloodstream-infection
MMH: Mnazi Mmoja Hospital
ESBL: extended-spectrum beta-lactamase
HIV: human immunodeficiency virus
PCR: polymerase chain reaction
